# Assessing School Engagement Intervention Dataset of Nigerian Pre-service TVET Teachers

**DOI:** 10.3389/fpsyg.2022.946001

**Published:** 2022-06-27

**Authors:** Godwin Keres Okoro Okereke, Samson Ikenna Nwaodo, Hyginus Osita Omeje, Joshua Onyedikachi Ike, Sylvanus Umunnakwe Njoku, George Nwachukwu Ogbonna, Victor Ikechukwu Oguejiofor, Ifeoma Bernadine Onah, Ogbonnaya Okorie Eze, Pauline Ijeoma Obe, Benedicta Anene Omeje, Ikechukwu Jerry Ogbonna, Nwahunanya Innocent, Veronica Nkechi Imakwu, Ogechukwu Onah, Catherine Chiugo Kanu, John Lliya, Ebiegberi Kontei, Eunice Nwakaego Onah

**Affiliations:** ^1^Faculty of Vocational and Technical Education, University of Nigeria, Nsukka, Nigeria; ^2^Department of Industrial Technology Education, Michael Okpara University of Agriculture, Umudike, Nigeria; ^3^Mechanical Engineering Department, Federal University of Technology, Owerri, Nigeria; ^4^Department of Technology and Vocational Education, Ebonyi State University, Abakaliki, Nigeria

**Keywords:** pre-service teachers, quantitative data, school engagement, Sidak, TVET schools, TVET teachers, Wilks' lambda

## Study Synopsis

It is a priority issue in technical and vocational education and training (TVET) to improve school engagement in order to promote teaching and learning in TVET institutions. This study appears to be the foremost to examine the dataset of a psychological intervention program for preservice TVET teachers' school engagement in a Nigerian public university. Using a school engagement measure, quantitative data was collected from 35 pre-service TVET teachers in a treatment group and 35 pre-service TVET teachers in a control group. To conduct the statistical analysis of the data from pre to posttest gathering, IBM SPSS version 22 was used. The study dataset showed that pre-service TVET teachers in the treatment condition demonstrated a significant increase in school engagement score at posttest compared to pre-service TVET teachers in the control condition. The technique used in this study to obtain the presented dataset may be very beneficial for understanding Nigeria's pre-service TVET teacher population when it comes to increasing their school engagement.

## Introduction

School engagement is a concept that encompasses student motivations, perceptions, and actions (Appleton et al., 2008; Baron and Corbin, 2012). The process of engagement may take several forms, such as participation, involvement, and integration, which require rational thinking and action. Over the past few years, the issue of school engagement in higher education has become increasingly important (Trowler, 2010). Because it provides a sense of belonging, a sense of purpose, academic success, and positive learning outcomes, school engagement is critical to the education system (Appleton et al., 2008; Furlong and Christenson, 2008; Dunleavy and Milton, 2009; Gunuc, 2013, 2014). Past studies indicate that most students enrolled in teacher training programs are moderately engaged at school (Gür, 2017; Ozdemir, 2017; Büyükgöze et al., 2018). In addition, study data of pre-service teachers show that those with higher academic achievement demonstrated a greater level of engagement than those with lower academic achievement (Gunuc, 2014; Bellici, 2015).

The term “pre-service teachers” refers to students who are enrolled in teacher education programs with the intent of gaining teaching credentials in order to teach in schools (Lee, 2019; Walker et al., 2021). The pre-service teacher must satisfy degree requirements, which include classroom work and field experience, before obtaining a teaching certificate (Lee, 2019). Pre-service TVET teachers are those students enrolled in a technical and vocational education and training (TVET) program at a higher education institution with no prior teaching experience. Critical thinking, solving complex problems, and data analysis and interpretation are crucial skills for these students. In the TVET curriculum, pre-service TVET teachers are equipped with the technical skills and information necessary for thriving in the profession they have chosen, and the workplace fundamentals (such as problem solving skills) necessary for career success (National Association of State Directors of Career and Technical Education, 2022). There have been many studies conducted on the subject of school engagement (Dunleavy and Milton, 2009; Gunuc, 2013; Kim and Corcoran, 2018). However, there has been only a little or no research done on the subject of school engagement in pre-service TVET teachers which means that there is limited data available to better understand this issue.

It is a priority issue in TVET to improve school engagement in order to promote teaching and learning in schools (Hui and Cheung, 2015; Jayalath and Esichaikul, 2020). As a result, we designed a psychological intervention program (i.e., a rational emotive behavior therapy program) to improve pre-service TVET teachers' school engagement in Nigeria and offer more insight into the effect of such a program through making the dataset publicly available. In the mid-50s, Albert Ellis founded rational emotive behavior therapy (REBT) as a psychological intervention program, which teaches people that their emotions are a result of their belief systems about the activating events, not the activating events themselves (Ellis, 1994). A central premise of REBT is that dysfunctional behaviors result from self-defeating thoughts (Ellis, 1995). According to Ogbuanya et al. (2019), REBT programs can be effective in addressing participants' problem behaviors, and their results can often be sustained for a long time after they are completed. There is a possibility that a REBT intervention can help students become more engaged in their schoolwork since there is a connection between attitudes toward school and school engagement (Ozdemir, 2017). Among adolescent students, an educational intervention incorporating REBT components significantly improved school engagement, as reported by Asogwa et al. (2020). Eseadi et al. (2022) demonstrates that REBT program is a significant type of psychological intervention that can be utilized to improve engagement at school, although their study focused on school workplace engagement of lecturers. It was therefore the purpose of this study to examine the dataset from a psychological intervention program (REBT program) for pre-service TVET teachers' school engagement in a Nigerian public university to determine the program's effectiveness.

## Methods

### Study Location and Participants

The study was conducted at a public university which is situated in the southeast region of Nigeria. A total of 70 pre-service TVET teachers were enrolled in the study, and all were low ranking in their cumulative grade point averages and did not show high school engagement. According to the randomization software (Saghaei, 2004), the study treatment condition consisted of 35 pre-service TVET teachers, while an active control condition consisted of 35 pre-service TVET teachers. A sample computation was performed using the GPower 3.1 software (Faul et al., 2007), the result of which showed a medium effect size 0.5 at a significance level of 0.05, a power of 85%, and a sample size of 22 as sufficient for this research. Participants' demographic data revealed that the average age of the participants was between the ages of 22 and 27 years old.

### Material

We used the School Engagement Scale (SES) (Fredricks et al., 2004) to collect the data for the study. Fredricks et al. (2004) developed the SES as a 19-item questionnaire that consists of cognitive, behavioral and emotional dimensions on a five-point scale of never (1) to all of the time (5). Increased levels of engagement were reflected in higher scores. The SES requires a minimum total school engagement score of 19 and a maximum total school engagement score of 95 to be considered engaged. Total engagement was calculated using the summated data of SES dimensions. The study classified participants' levels of SES engagement into three categories: low engagement (30 points), 30 points, and 60 points (Asogwa et al., 2020). In prior studies, the school engagement scale was found to be reliable (Fredricks et al., 2004; Asogwa et al., 2020). In the current study, the SES's internal consistency reliability was 0.923.

### Procedure

The participants in this study were those with a low SES score at pretest. A written informed consent was required from potential participants, along with proof of enrollment in a TVET higher education program. Pre-service TVET teachers received information about the study via email, word-of-mouth and posters. The REBT intervention designed to help pre-service TVET teachers become more engaged in school was adapted from Asogwa et al. (2020). As part of the intervention, topics on school engagement included a definition of school engagement and its significance, school activities and engagement, school engagement and relationships with peers, staff, parents, academic achievement and life after graduation, risks associated with low school engagement, and ways to maintain high levels of engagement. Participants were given 20 min for reflection, asking questions, and receiving feedback. Participants and therapists worked together on various therapeutic tactics, including disputing, positive reinforcement, cognitive rehearsal, and role-playing. Each session of REBT ended with homework assignments. Cognitive restructuring was used to improve homework completion. This REBT intervention consisted of eight treatment sessions over 8 weeks. A 60-min session was held for each of the treatment sessions. During the validating of the program's content, three therapists provided assistance.

The IBM Statistical Package for the Social Sciences was the primary statistical application used in data processing and analysis (IBM SPSS, version 22). The repeated measures multivariate analysis of variance was used to analyze the data at the 0.05 probability level. The Wilks' lambda (Λ) and Partial η^2^ have both been reported as indicators of study effects. In addition, we conducted Sidak's *post-hoc* test of pre-service TVET teachers' school engagement data by group x time interaction to determine which group experienced improvement.

## Results and Discussion

**Figure 1:**
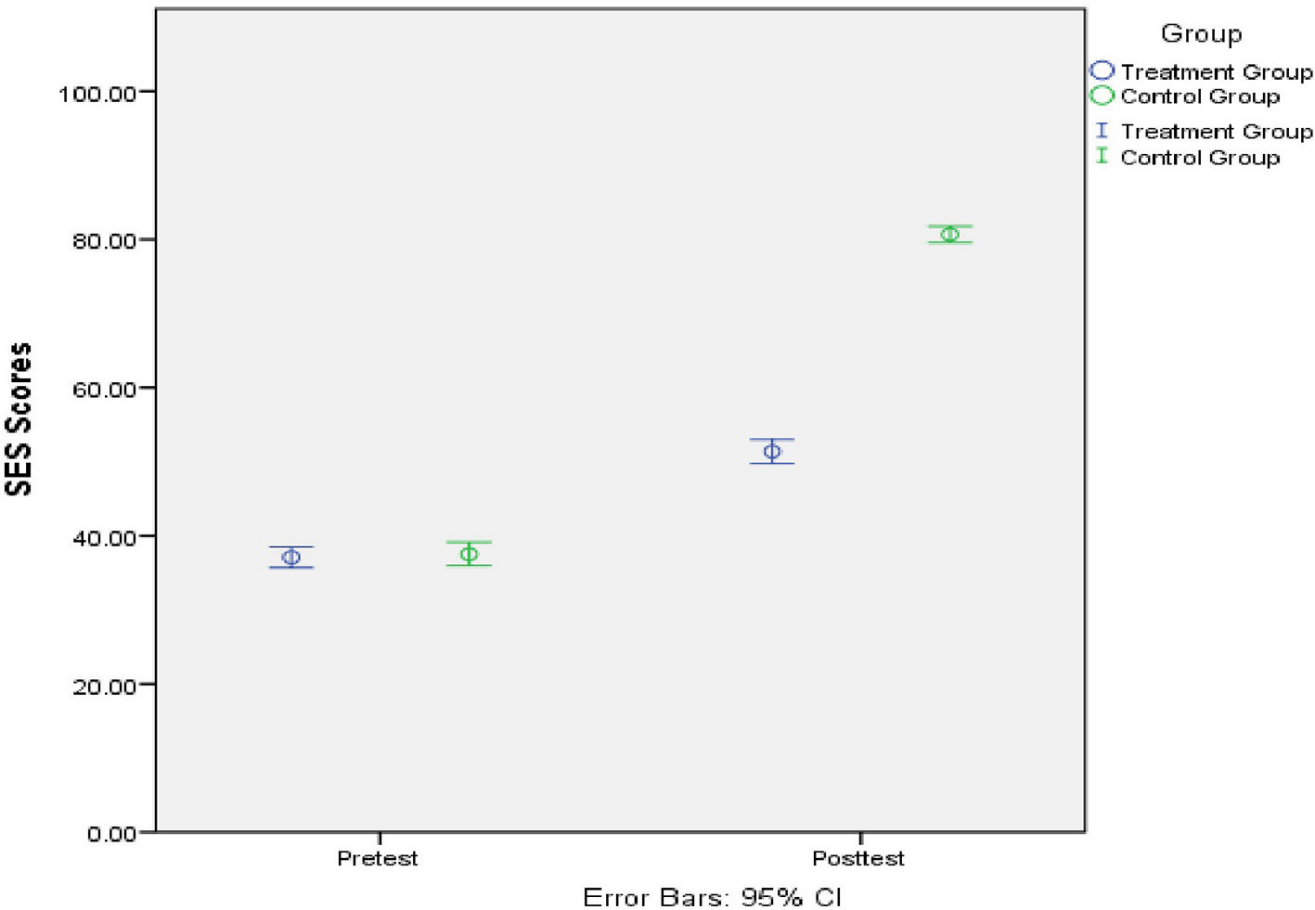
Pre-service TVET teacher's school engagement scores by group.

Table 1 presents the descriptive statistics of the dataset collected from each of the study groups. The pretest data indicated that there was no significant difference when comparing the school engagement of pre-service TVET teachers in the treatment group and the control group [*F*_(1, 69)_ = 0.170, *p* = 0.681, Partial η^2^ = 0.002]. The posttest data showed that REBT program contributed significantly to the improvement of pre-service TVET teachers' school engagement in the treatment group compared with the control group [*F*_(1, 69)_ = 921.08, *p* = 0.000, Partial η^2^ = 0.931]. The data processing with multivariate tests indicated that the REBT program had significant beneficial effects on the pre-service TVET teachers' school engagement [*F*_(2, 67)_ = 463.73, *p* = 0.000, Partial η^2^ = 0.933, Wilks' Λ = 0.067]. With Sidak's *post-hoc* procedure for the Group × Time interaction, the pretest data for pre-service TVET teachers' school engagement in the treatment group was shown to be significantly similar to that of the control group (*Mean difference* = −0.43, standard error = 1.04, *p* = 0.681, 95%CI: −2.50,1.64). However, the posttest school engagement data from pre-service TVET teachers in the treatment group demonstrated higher school engagement than that from pre-service TVET teachers in the control group (Mean difference = −29.29, standard error = 0.97, *p* = 0.000, 95%CI: −31.21, −27.36). Thus, there was a significant improvement in the school engagement of pre-service TVET teachers in the treatment group compared with those in the control group as shown by the study data (see Figure 1). The presented data is consistent with those of an educational intervention incorporating REBT components that increased adolescent students' school engagement (Asogwa et al., 2020). The data is also consistent with those of previous study which discovered that REBT had a significant beneficial effect on reducing problem behavior in students (Ezeribe, 2019). According to Onuigbo et al. (2018), the REBT intervention group experienced a significant decrease in school-related irrational thinking at posttest in their study. A REBT intervention data showed it was effective in increasing students' resilience and decreasing their school-related anxiety in a previous research (Noormohamadi et al., 2022). According to Eremie et al. (2020), when undergraduate students were compared to a control group, REBT treatment components resulted in a significant improvement in students' self-concept.

**Table 1:** Descriptive statistics of data collected from each of the study groups.

**Data type**	**Group**	**Mean**	**SD**	**Kurtosis**	**Skewness**
Pretest data	Control	37.09	4.09	−1.38	0.05
	Treatment	37.51	4.58	−1.00	0.14
Posttest data	Control	51.37	4.72	−0.59	−0.19
	Treatment	80.66	3.20	0.98	1.13

*SD, Standard deviation*.

The first limitation of the study is that it focused exclusively on pre-service TVET teachers at a single public university. To make generalizations, additional research in other public universities, as well as comparisons among universities regarding pre-service TVET teachers' school engagement, may be conducted. The second limitation is the sample size. A larger sample size may have been used at the university to increase the population's representativeness. Additionally, because pre-service teachers in different departments may have varying levels of school engagement, it is suggested that a study be conducted to assess pre-service teachers' levels of school engagement and commitment to teaching in relation to their respective programs. Additionally, because this was a quantitative study, certain limitations may have occurred as a result of the nature of such studies. Qualitative research would be required to deeply ascertain the extent to which pre-service TVET teachers are engaged. Qualitative research techniques, such as conducting interviews or holding focus groups, may yield more precise and student-specific variables. Given the significance of school engagement in higher education and the paucity of research on school engagement among pre-service TVET teachers, such research could shed light on an often-overlooked aspect of pre-service TVET teachers' commitment to teaching and assist policymakers, researchers, and trainers in effectively addressing teacher shortages and high turnover rates.

## Conclusion

The current research examined the dataset obtained from a school engagement intervention among a sample of Nigerian pre-service TVET teachers. The study dataset showed that REBT program is a significant type of intervention that can increase school engagement of pre-service TVET teachers. According to the current study's dataset, when compared to a control group, the REBT intervention increased pre-service TVET teachers' school engagement.

## Data Availability Statement

The original contributions presented in the study are included in the article/supplementary material, further inquiries can be directed to the corresponding author/s.

## Ethics Statement

Ethics approval for this study was obtained from the Committee on Research Ethics (Faculty of VTE Research Ethics Committee) at the authors' institution (University of Nigeria). The patients/participants provided their written informed consent to participate in this study.

## Author Contributions

GKOO, SIN, HO, JI, SUN, GNO, and VO conceived the study. GKOO, SIN, HO, JI, SUN, GNO, VO, IO, OE, PO, BO, IO, NI, VN, OO, CK, JL, EK, and EO designed the research methodology and carried out the study equally. All authors agree to be accountable for the content of the work. All authors contributed to the article and approved the submitted version.

## Conflict of Interest

The authors declare that the research was conducted in the absence of any commercial or financial relationships that could be construed as a potential conflict of interest.

## Publisher's Note

All claims expressed in this article are solely those of the authors and do not necessarily represent those of their affiliated organizations, or those of the publisher, the editors and the reviewers. Any product that may be evaluated in this article, or claim that may be made by its manufacturer, is not guaranteed or endorsed by the publisher.
